# Malignant Perivascular Epithelioid Cell Tumor Originating from the Transverse Mesocolon

**DOI:** 10.70352/scrj.cr.26-0592

**Published:** 2026-10-28

**Authors:** Ryo Yokota, Ryo Ohta, Kohki Takeda, Kumiko Sekiguchi, Takao Shimizu, Norio Motoda, Nobuhiko Taniai, Akihisa Matsuda

**Affiliations:** 1 Department of Gastroenterological Surgery, Nippon Medical School Musashikosugi Hospital, Kawasaki, Kanagawa, Japan; 2 Department of Diagnostic Pathology, Nippon Medical School Musashikosugi Hospital, Kawasaki, Kanagawa, Japan; 3 Department of Gastroenterological Surgery, Nippon Medical School, Tokyo, Japan

**Keywords:** perivascular epithelioid cell tumors (PEComas), malignant perivascular epithelioid cell tumors, mesentery, transverse mesocolon, surgical resection

## Abstract

**INTRODUCTION:**

Perivascular epithelioid cell tumors (PEComas) are rare mesenchymal neoplasms with melanocytic and smooth muscle differentiation. Primary mesenteric PEComas are exceptionally uncommon and usually cannot be diagnosed from imaging alone. We report a case of malignant PEComa arising from the transverse mesocolon.

**CASE PRESENTATION:**

A 34-year-old woman was referred to our department after abdominal ultrasonography showed a large intra-abdominal mass. Contrast-enhanced CT revealed a 15-cm heterogeneous, hypervascular mesenteric tumor with internal necrosis and feeding vessels from the superior mesenteric artery. MRI showed no continuity with the uterus or ovaries. Because percutaneous biopsy was considered to carry a substantial risk of bleeding and dissemination, the tumor was resected for diagnosis and treatment. Laparotomy demonstrated a transverse-mesocolic tumor directly invading the transverse colon; transverse colectomy with en bloc tumor resection was performed. Histopathological evaluation showed pleomorphic epithelioid cells, marked nuclear atypia, coagulative necrosis, vascular invasion, and frequent mitoses. The tumor was positive for HMB-45, Melan-A, and smooth muscle actin and negative for TFE3, supporting malignant PEComa. The postoperative course was uneventful, and the patient remained free of recurrence 4 months after surgery.

**CONCLUSIONS:**

Malignant PEComa of the transverse mesocolon is extremely rare. This tumor should be considered among large, hypervascular mesenteric tumors, although imaging is not specific. Complete resection with negative margins is the principal treatment for localized disease. High-risk pathological features justify prolonged cross-sectional imaging surveillance and multidisciplinary, molecularly informed management if recurrence develops.

## Abbreviations

AML
: angiomyolipoma
DWI
: diffusion-weighted imaging
GIST
: gastrointestinal stromal tumor
HMB-45
: human melanoma black-45
HPF
: high-power field
mTOR
: mammalian target of rapamycin
PEComa
: perivascular epithelioid cell tumor
SFT
: solitary fibrous tumor
SMA
: smooth muscle actin
TFE3
: transcription factor E3
TSC
: tuberous sclerosis complex

## INTRODUCTION

PEComas are rare mesenchymal neoplasms comprised of perivascular epithelioid cells that show melanocytic and smooth muscle differentiation. The PEComa family includes AML, lymphangioleiomyomatosis, clear cell “sugar” tumor, and PEComa not otherwise specified.^1–3)^ Although PEComas may arise from various sites such as the uterus, retroperitoneum, gastrointestinal tract, and soft tissues, primary mesenteric PEComas are exceptionally rare.

PEComas range from benign to overtly malignant. No symptom or imaging feature reliably differentiates PEComa from other mesenchymal or gastrointestinal tumors; diagnosis therefore depends on histopathological and immunohistochemical assessment.^4,5)^ Because standardized treatment and surveillance strategies for malignant mesenteric PEComa have not been established, the details of resection, pathological risk, recurrence patterns, and practical follow-up are clinically relevant. We report a malignant PEComa arising from the transverse mesocolon in a young woman and discuss these considerations.

## CASE PRESENTATION

A 34-year-old woman who had no relevant medical or family history consulted a local clinic with a palpable lower abdominal mass. Abdominal ultrasonography showed an approximately 10-cm intra-abdominal mass, and she was referred to our department for further assessment and treatment. Physical examination confirmed the presence of a lower abdominal mass. She had no abdominal pain, fever, bowel obstruction, or other systemic symptoms. Laboratory testing revealed mild anemia (hemoglobin, 10.5 g/dL); other results were unremarkable. Serum carcinoembryonic antigen, carbohydrate antigen 19-9, lactate dehydrogenase, and soluble interleukin-2 receptor concentrations were within their reference ranges.

Contrast-enhanced CT showed a 15-cm mesenteric mass with heterogeneous enhancement and a low-attenuation area suggestive of internal necrosis (**Fig. 1A**). 3D vascular reconstruction demonstrated feeding vessels branching from the superior mesenteric artery, supporting a mesenteric origin (**Fig. 1B**); no distant metastasis was detected. MRI revealed that the mass was distinct from the uterus and ovaries (**Fig. 1C**). DWI showed high signal within the tumor (**Fig. 1D**); because an apparent diffusion coefficient map was not available, restricted diffusion could not be confirmed, and the finding was regarded as nonspecific. Esophagogastroduodenoscopy showed no abnormality. During colonoscopy, the scope could not be advanced beyond the descending colon because of extrinsic compression by the mass; no mucosal lesion was visible in the examined colon.

**Fig. 1:**
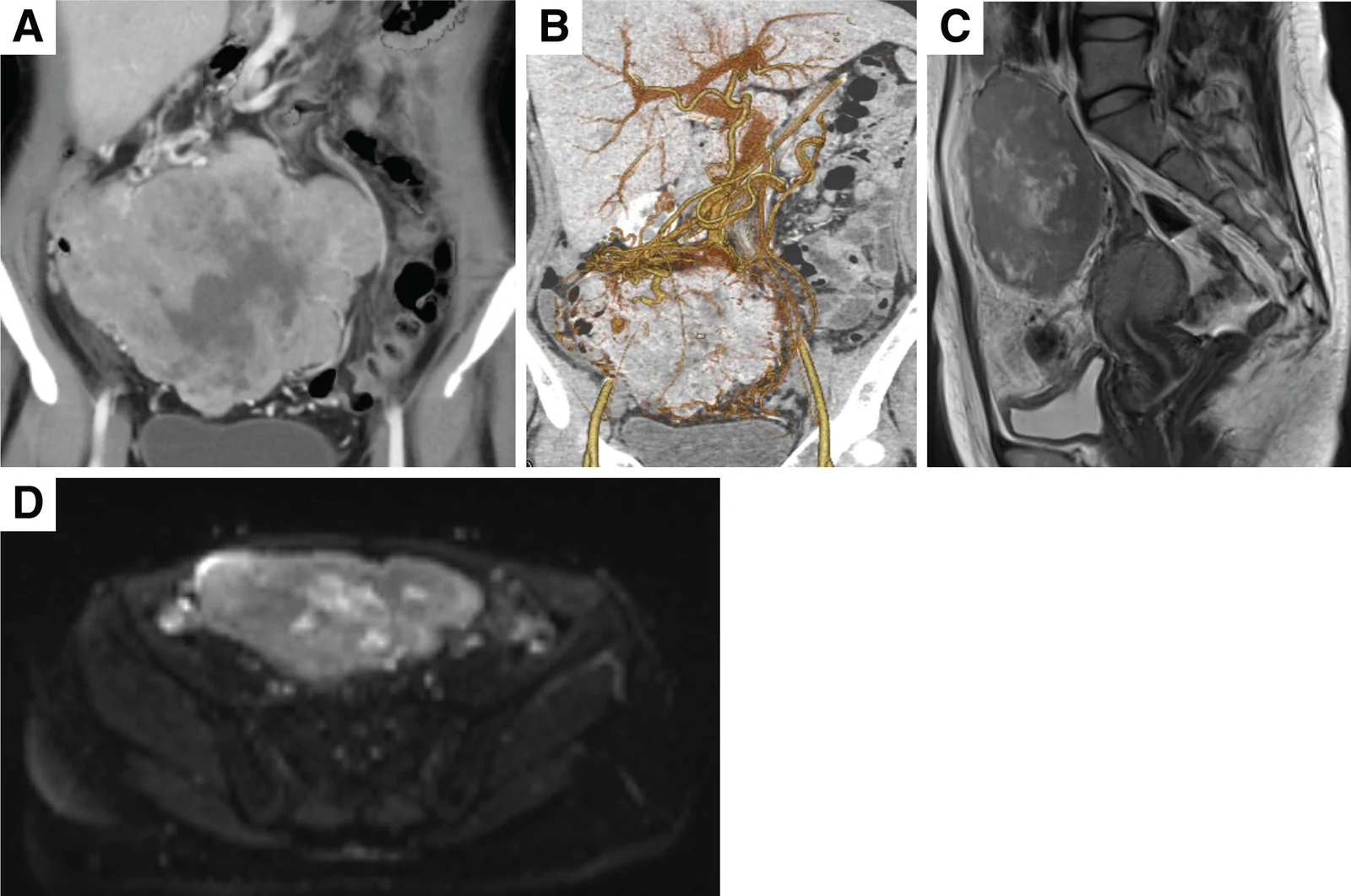
Preoperative imaging findings. (**A**) Contrast-enhanced CT showing a large heterogeneous mass in the lower abdomen with a central low-attenuation area. (**B**) 3D vascular reconstruction showing feeding vessels arising from the superior mesenteric artery. (**C**) T2-weighted MRI showing that the tumor is separate from the uterus and ovaries. (**D**) DWI showing high signal within the tumor. An apparent diffusion coefficient map was unavailable. DWI, diffusion-weighted imaging

Percutaneous needle biopsy was considered but not performed because the large, hypervascular tumor with suspected necrosis posed risks of bleeding and possible peritoneal dissemination. Because malignancy could not be excluded and the diagnosis remained uncertain, surgical resection was undertaken for both diagnosis and treatment.

At laparotomy, the tumor was centered in the transverse mesocolon (**Fig. 2A**). No ascites, peritoneal dissemination, liver metastasis, or distant metastasis was present. The tumor directly invaded the transverse colon but no other organ; therefore, transverse colectomy with en bloc resection of the mesocolic tumor was performed. The operative time was 162 minutes, and the estimated blood loss was 10 g. The resected specimen measured approximately 20 × 17 × 6.5 cm. The mucosal surface showed focal exposure of the tumor with central necrosis (**Fig. 2B**), and the cut surface showed a solid, gray-white, medullary mass with necrotic areas (**Fig. 2C**). All microscopic proximal, distal, and mesenteric/radial resection margins were negative, establishing an R0 resection.

**Fig. 2:**
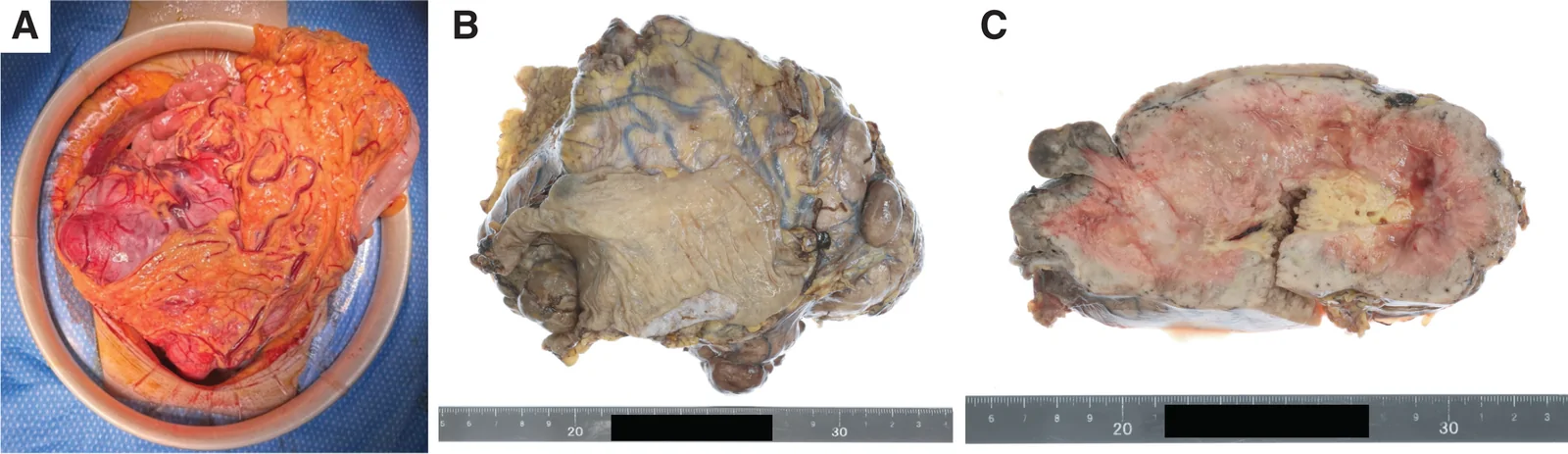
Intraoperative and gross pathological findings. (**A**) Intraoperative view of the tumor arising from the transverse mesocolon. (**B**) Mucosal surface of the resected transverse colon showing focal tumor exposure and central necrosis. (**C**) Cut surface showing a solid gray-white medullary mass with necrotic areas.

Histologically, atypical pleomorphic polygonal-to-epithelioid cells with enlarged nuclei and eosinophilic cytoplasm proliferated in solid sheets around variably sized vessels. Coagulative necrosis, frequent mitotic figures including atypical mitoses, and vascular invasion were present (**Fig. 3**). On serial microscopic examination, the tumor epicenter was within the transverse mesocolon, and the tumor extended contiguously into the mucosal surface of the transverse colon, with no separate intramural primary focus; these findings supported a primary transverse-mesocolic origin with direct colonic invasion. Immunohistochemically, the tumor cells were positive for HMB-45, Melan-A, and SMA, with focal or variable positivity for S-100, c-kit, CD34, and CD31; DOG1 and TFE3 were negative (**Fig. 4**). Although c-kit and CD34 staining raised the possibility of GIST, the morphology, strong melanocytic-marker expression, SMA expression, and lack of DOG1 supported PEComa. Negative TFE3 immunostaining did not support a TFE3-associated subset, but TFE3 rearrangement was not assessed molecularly and therefore was not formally excluded. The tumor met the Folpe criteria for malignant PEComa because it showed 2 or more adverse features: size >5 cm, infiltrative growth, high nuclear grade and cellularity, mitotic activity >1/50 HPF, necrosis, and vascular invasion.^5)^

**Fig. 3:**
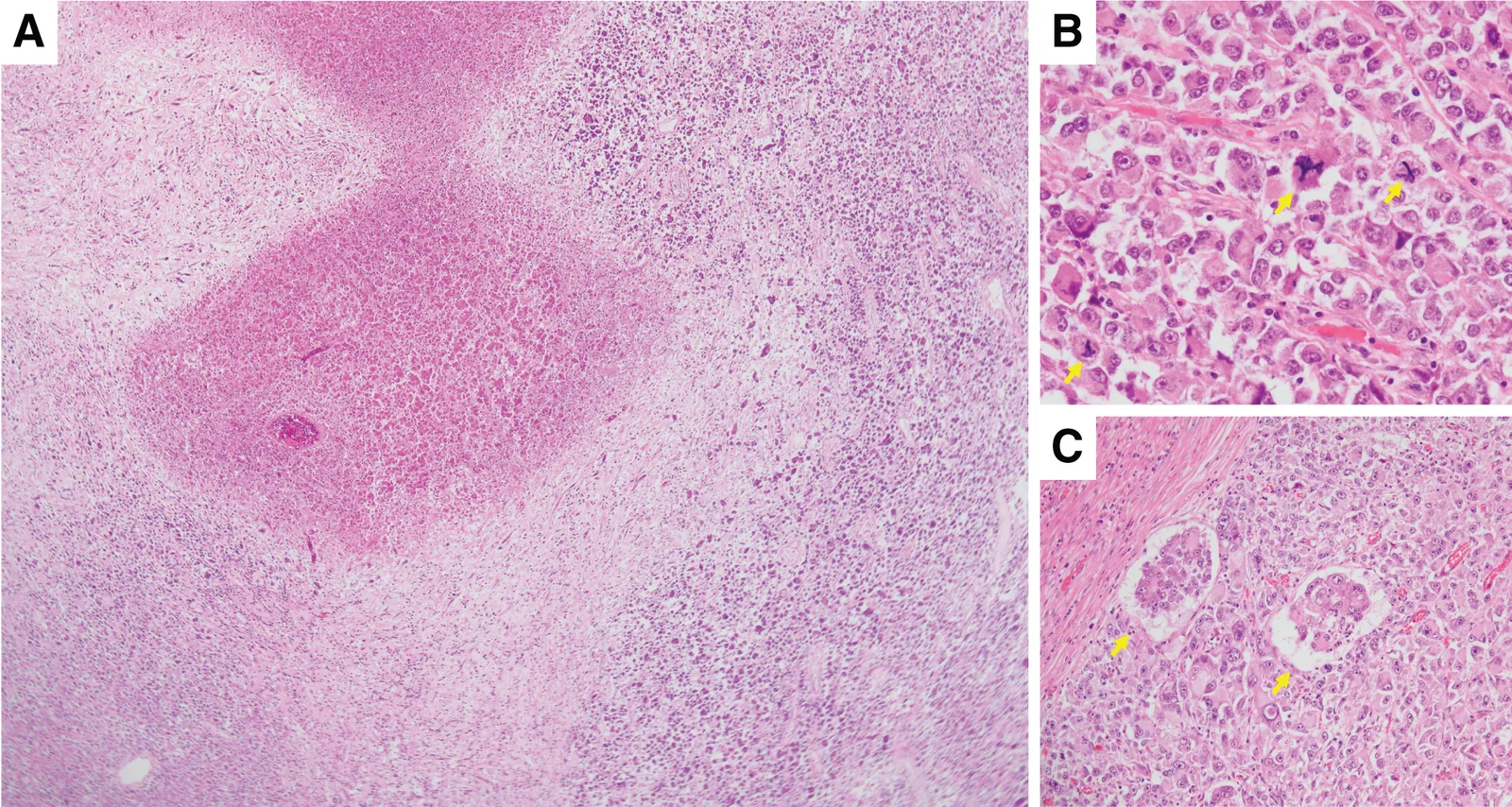
Histopathological findings. (**A**) Coagulative necrosis (hematoxylin and eosin staining; original magnification, ×1.25). (**B**) Atypical pleomorphic epithelioid cells with enlarged nuclei and eosinophilic cytoplasm growing in a solid pattern. Numerous mitotic figures, including atypical mitoses (arrows), are present (hematoxylin and eosin staining; original magnification, ×40). (**C**) Vascular invasion by tumor cells (hematoxylin and eosin staining; original magnification, ×20).

**Fig. 4:**
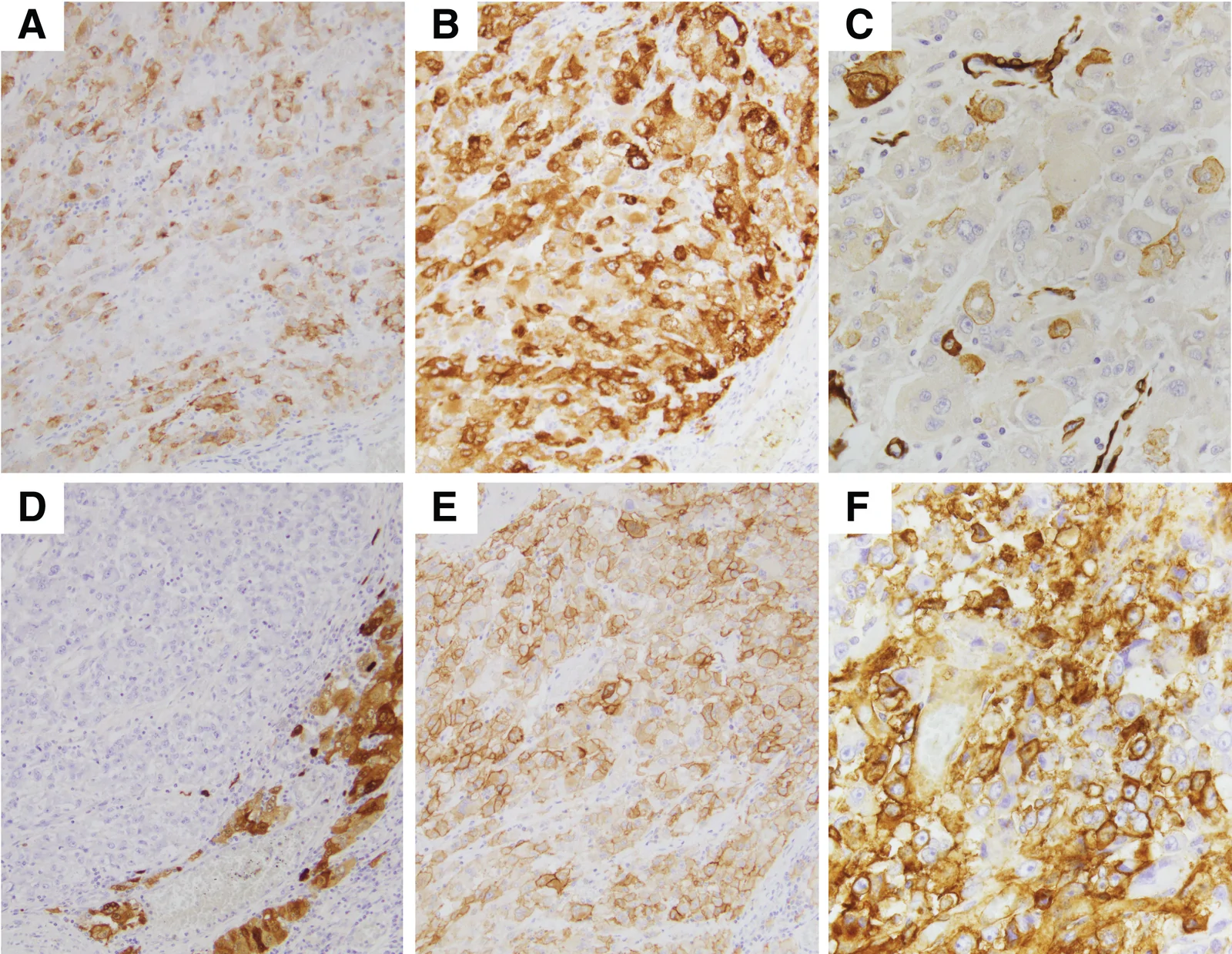
Immunohistochemical findings. Tumor cells were positive for HMB-45 (**A**), Melan-A (**B**), and SMA (**C**), with focal or variable positivity for S-100 (**D**), c-kit (**E**), and CD34 (**F**) (original magnification, ×20 for all panels). SMA, smooth muscle actin

The postoperative course was uneventful, and the patient was discharged on POD 7. No adjuvant therapy was administered after complete resection because evidence supporting adjuvant systemic therapy for PEComa is lacking. At 4 months after surgery, the patient remained well without evidence of recurrence or metastasis. The current surveillance plan is contrast-enhanced CT of the chest, abdomen, and pelvis every 3 months for the first 2 years, every 6 months during years 3–5, and annually thereafter through year 10, together with clinical assessment and laboratory testing at each visit.

## DISCUSSION

PEComa is defined by a characteristic morphology and coexpression of melanocytic and smooth muscle markers, but its imaging appearance is variable.^2,3,6,7)^ A valid percentage of PEComa among all intra-abdominal tumors cannot be calculated because population-based, site-specific denominators are unavailable and published data consist largely of small series and case reports. Malignant PEComa is classified among ultra-rare sarcomas. However, the proportion of PEComas among all intra-abdominal tumors and the population incidence of primary mesenteric PEComa have not been established.^8)^ A focused review through August 2026 identified 16 previously reported patients whose tumors were explicitly described as primary mesenteric or mesocolic PEComa or PEComa not otherwise specified (**Table 1**).^5,9–20)^ Reports that did not establish a primary mesenteric origin and reports of conventional AML were excluded to avoid mixing distinct anatomic or pathological entities. The small number of cases and substantial missing data preclude reliable estimates of sex distribution, recurrence rate, or prognosis.

**Table 1:** Published primary mesenteric or mesocolic PEComas

**Reference**	**Age, y/sex**	**Site**	**Size, cm**	**Key pathological findings**	**Disease at presentation/resection**	**Posmiddleerative recurrence**	**Last reported status**
Folpe et al.^5)^	67/F	Mesentery	13	UMP; necrosis, no; 0 MF/50 HPF; invasion, no	Localized; SE	No	NED, 84 months
Folpe et al.^5)^	97/F	Mesentery	4	PEComa; necrosis, no; 0 MF/50 HPF; invasion, no	Localized; SE	No	NED, 38 months
Folpe et al.^5)^	80/F	Mesentery	9.5	Malignant; necrosis, yes; >50 MF/50 HPF; vascular invasion	Localized; SE	No	NED, 19 months
Folpe et al.^5)^	46/F	Mesentery	12	Malignant; necrosis, yes; 5 MF/50 HPF; vascular invasion	Localized; SE + chemotherapy	Yes, 22 months; intra-abdominal recurrence and liver metastases; modality, NR	Died, 27 months
Gross et al.^9)^	5.5/M	Ascending-colon mesentery	5	PEComa; necrosis, no; MF, NR; surrounding-tissue invasion	Localized; SE	No	NED, 24 months
Lai et al.^10)^	59/M	Jejunal mesentery	11	Malignant; necrosis, yes; 3 MF/50 HPF; vascular invasion	Localized; SE + chemoradiotherapy	Yes, 6 months; abdominopelvic; CT	Alive; duration, NR
Fu et al.^11)^	38/F	Jejunal mesentery	10	Malignant; necrosis, yes; 2 MF/50 HPF; jejunal muscular invasion; no vascular invasion	Regional mesenteric LN involved; total resection + chemotherapy; postoperative PET/CT negative	No	NED, 6 months
Kapur et al.^12)^	49/F	Mesocolon near the hepatic flexure	13.5	Malignant; necrosis, yes; extensive lymphovascular invasion; large- and small-bowel invasion; MF count, NR	Liver and peritoneal metastases at presentation; palliative bowel/tumor resection; everolimus	Not applicable: metastatic at presentation	Response to everolimus reported; duration, NR
Wejman et al.^13)^	67/F	Sigmoid mesentery	3.5	PEComa; necrosis, no; 1–2 MF/50 HPF; no vascular invasion	Localized; radical resection	NR	PEComa-specific follow-up, NR
Shi et al.^14)^	48/F	Small-bowel mesentery	14	Malignant; necrosis, yes; elevated MF; no vascular invasion	Localized; SE + oxaliplatin	No	NED, 60 months
Hasan et al.^15)^	27/M	Terminal-ileum mesentery	4	UMP (original diagnosis); necrosis, no; 2 MF/50 HPF; vascular invasion not explicitly reported	Localized; complete resection	No	NED, >3 y
Kim et al.^16)^	50/F	Rectal mesentery	8	Malignant; necrosis, yes; 40 MF/50 HPF; surrounding-tissue invasion	Localized; SE + chemotherapy	No	NED, 6 months
Jindal et al.^17)^	Young/F; exact age UA	Mesentery	UA	PEComa; details, UA	Para-aortic nodal metastasis reported; initial extent and operative details UA	Temporal relation to primary surgery, UA; detection modality, UA	UA
Meredith et al.^18)^	26/M	Transverse-colon mesentery	23	Malignant; necrosis, yes; >100 MF/50 HPF	Multiple peritoneal metastases at diagnosis; palliative R2 resection	Not applicable (metastatic at presentation)	Stable at 1 months on nab-sirolimus; progression after 2 cycles with treatment breaks; gemcitabine/docetaxel, later with sirolimus; final follow-up, NR
Al-Nahhas et al.^19)^	30/F	Mesentery	7.5	Benign; necrosis, no; MF, absent; invasion, no	Localized; SE	NR	NR
Sridhar et al.^20)^	14/M	Mesentery of the hepatic flexure	6.7	Benign (original diagnosis); no atypia, mitoses, or lymphovascular invasion; gross focal necrosis, absent in illustrated histology	Localized; biopsy, then margin-negative excision	No local recurrence on US at 24 months	Healthy, 24 months
Present case	34/F	Transverse mesocolon	20	Malignant; necrosis, yes; 7–10 MF/50 HPF; vascular and colonic invasion	Localized; R0 resection	No	NED, 4 months

Pathological classifications follow the original reports. Postoperative recurrence is distinguished from metastases present at initial diagnosis and from progression of residual disease. UA does not imply that the full article omitted the information. For Sridhar et al., gross focal necrosis was described, whereas the illustrated histology showed no necrosis. Reports without an established primary mesenteric origin and conventional AMLs were excluded. Reference numbers correspond to the main text.

AML, angiomyolipoma; F, female; HPF, high-power field; LN, lymph node; M, male; MF, mitotic figures; NED, no evidence of disease; NR, not reported; PEComas, perivascular epithelioid cell tumors; R0, microscopically margin-negative resection; R2, macroscopic residual disease; SE, surgical excision; UA, unavailable in the accessible abstract (Jindal et al.); UMP, uncertain malignant potential; US, ultrasonography; y, years

The preoperative differential diagnosis in this case included exophytic or extra-gastrointestinal GIST, lymphoma, schwannoma, leiomyoma, desmoid-type fibromatosis, SFT, myelolipoma, and sarcomas such as leiomyosarcoma and liposarcoma.^6,7,21)^ GIST may grow exophytically and show heterogeneous enhancement, hemorrhage, or necrosis; however, no convincing bowel-wall epicenter was identified, which argued against but did not exclude GIST. Lymphoma was less favored because the lesion was markedly heterogeneous and hypervascular with necrosis, there was no lymphadenopathy, and lactate dehydrogenase and soluble interleukin-2 receptor concentrations were normal. Schwannoma and leiomyoma may be circumscribed and can show degeneration and high signal on DWI, so MRI alone could not exclude them. A desmoid tumor may arise in the mesentery, but prominent hypervascularity and central necrosis were atypical for a fibrous desmoid lesion. The absence of macroscopic fat argued against myelolipoma and classic AML. SFT may be a well-circumscribed, intensely enhancing mass with prominent feeding vessels and can be radiologically indistinguishable from PEComa. The large size, heterogeneous enhancement, and necrosis were also compatible with a high-grade sarcoma, including leiomyosarcoma; liposarcoma was less likely because no macroscopic fat was present. Thus, imaging narrowed the differential and demonstrated resectability but did not specifically diagnose PEComa.

Histopathology established both the diagnosis and the transverse mesocolic origin. The tumor displayed perivascular epithelioid morphology, melanocytic-marker expression, and smooth muscle differentiation, while DOG1 negativity weighed against GIST. The mesocolic epicenter and contiguous invasion of the adjacent colon supported origin in the transverse mesocolon rather than a primary intramural colonic tumor. TFE3 immunohistochemistry was negative; because immunohistochemistry is not equivalent to a rearrangement assay, the result was interpreted as not supporting, rather than excluding, a TFE3-rearranged PEComa.

For localized PEComa, complete surgical resection with negative microscopic margins remains the principal potentially curative treatment. Evidence does not establish a role for adjuvant systemic therapy after R0 resection. For unresectable or metastatic malignant PEComa, mTOR-pathway inhibition has the strongest prospective evidence: in the phase II AMPECT study, nab-sirolimus achieved an objective response in 39% of 31 evaluable patients, with especially frequent responses in tumors harboring TSC2 alterations.^22)^ Earlier reports also documented activity of oral sirolimus in malignant PEComa.^23)^ If this patient develops an isolated, technically resectable recurrence, a repeat complete resection would be discussed by a multidisciplinary sarcoma team. For unresectable, multifocal, or metastatic recurrence, the tumor would undergo molecular profiling, including assessment of TSC1/TSC2 pathway alterations and TFE3 rearrangement, and mTOR-inhibitor–based therapy would be considered according to drug access, regulatory status, and patient factors. Local ablation or radiotherapy may be considered for selected oligometastatic or symptomatic sites. Conventional sarcoma chemotherapy has limited evidence and would be individualized after specialist consultation or within a clinical trial.

**Table 1** distinguishes metastatic disease at presentation from postoperative recurrence. The case reported by Hasan et al. remained disease-free for more than 3 years and was not a recurrence.^15)^ Postoperative recurrence was reported in a 46-year-old woman in the Folpe series (intra-abdominal recurrence and liver metastases at 22 months) and the patient reported by Lai et al. (abdominopelvic recurrence detected by CT at 6 months).^5,10)^ Jindal et al. reported para-aortic nodal metastasis; however, its temporal relationship to primary surgery and the detection modality could not be established from the accessible abstract.^17)^ By contrast, the patient described by Fu et al. had regional nodal involvement at presentation, and the patient described by Meredith et al. had multiple peritoneal metastases at presentation; neither finding should be counted as postoperative recurrence.^11,18)^ Because late recurrence is possible and many reports have short or unreported follow-up, prolonged cross-sectional imaging surveillance is reasonable. Our practical schedule emphasizes the first 2 years but continues through at least 10 years; the interval may be modified according to new findings, radiation exposure, renal function, and multidisciplinary review.

## CONCLUSIONS

We report a malignant PEComa arising from the transverse mesocolon and directly invading the transverse colon. PEComas should be considered in the differential diagnosis of a large, hypervascular mesenteric mass, although imaging is nonspecific and definitive diagnosis requires pathological evaluation. R0 resection is the preferred treatment for localized disease. The combination of large size, infiltrative growth, high-grade cytology, mitotic activity, necrosis, and vascular invasion supports consideration of prolonged CT surveillance; resectable recurrence should prompt consideration of repeat local treatment, whereas unresectable disease should prompt molecular profiling and mTOR-inhibitor–based multidisciplinary management.

## DECLARATIONS

### Use of generative artificial intelligence (AI) and AI-assisted technologies in the writing process

During preparation of this manuscript, the authors used OpenAI for English language editing and editorial revision support. The authors reviewed and edited the output and took full responsibility for the content of the manuscript.

### Availability of data and materials

The datasets supporting the conclusions of this article are included within the article. Additional data are not publicly available because of patient privacy concerns but are available from the corresponding author on reasonable request.
